# Reducing the pollutant load of olive mill wastewater by photocatalytic membranes and monitoring the process using both tyrosinase biosensor and COD test

**DOI:** 10.3389/fchem.2013.00036

**Published:** 2013-12-16

**Authors:** Elisabetta Martini, Mauro Tomassetti, Luigi Campanella, Antonio Fortuna

**Affiliations:** ^1^Department of Chemistry, “Sapienza” University of RomeRome, Italy; ^2^SE.TE.C. SRLCivita Castellana, Italy

**Keywords:** olive mill wastewater (OMW), photocatalysis, photoreactor, tyrosinase biosensor, COD

## Abstract

Photocatalytic technique had already been employed in the treatment of olive mill wastewater (OMW) using the photocatalysis in suspension. The coupling of photocatalytic and membrane techniques should result in a very powerful process bringing great innovation to OMW depollution. Despite the potential advantages using these hybrid photoreactors, research on the combined use of photocatalysis and membranes has so far not been sufficiently developed. The present paper describes a study to assess the photocatalytic efficacy of a new ceramic membrane containing titanium dioxide, irradiated by UV light, used to abate the pollutant load of OMW. Good results were obtained (more than 90% of the phenol content was removed and the COD decrease was of the order of 46–51% in 24 h) particularly using the ceramic membrane compared with those offered by analogous catalytic membranes made of metallic or polymeric materials.

## Introduction

There are literature reports of a good number of studies (Saez et al., 1992; Paraskeva et al., 2004; Dhaouadia and Marrot, 2008) on the possibility of abating or reducing the pollutant load of olive mill wastewater (OMW). The olive oil extraction process generates large amounts of dark liquid effluent known as OMW, as high as 0.5–0.8 m^3^ per tonne of olives treated. This effluent consists of a mixture of “vegetation water” coming from the olives, and water added during oil extraction process. OMW is one of the most contaminated effluents. OMW is a foul smelling acidic wastewater, composed of water (83–92 W/W%), organic matter (4–16 W/W%) and minerals (1–2 W/W%). OMW disposal on farmland is the cause of serious problems owing to the wastewater's high phenol and polyphenol content.

Owing to their antibacterial effects, phenols and polyphenols are the most problematic compounds contained in the OMW (Saez et al., 1992; Pozzo et al., 1997). Alternative processes have been proposed to reduce pollutant problems such as those due to OMW. According to the literature, the proposed OMW treatment processes can be physical-chemical, biological or combined treatment (Boari and Mancini, 1990; Fiestas De Ursinos and Padilla, 1992; Pozzo et al., 1997; Paraskeva and Diamadopoulos, 2006). Treatment of OMW by advanced oxidation processes (AOPs), such as electrochemical oxidation, has recently been increasing (Andreozzi et al., 1999; Drouiche et al., 2004; Inan et al., 2004; Gotsi et al., 2005; Giannis et al., 2007). Recent studies used solar photocatalytic pilot plants to obtain the OMW degradation by combined TiO_2_ (very widely used also in the degradation of textile reactive dye) and photo-Fenton catalysis system (Ahmadi et al., 2005). The present research therefore aims at investigating the catalytic effect of titanium dioxide, together with the action of ultraviolet light and the oxidizing effect of hydrogen peroxide. The tests were performed in a photoreactor in which three different types of heterogeneous membrane containing titanium dioxide were successively inserted, one at a time. Of these three different membranes one was ceramic, one polymeric and the third metallic. Significant advances in membrane technology research aimed at extending membrane potential have recently been reported.

Numerous membrane applications have also been proposed that are regarded as economically competitive due to the availability of membranes with higher flux and lower process costs. In particular, ceramic membrane development has been one of the principal targets. These membranes have proved suitable for high temperature, corrosive and high pressure applications with good durability (DeFriend et al., 2003; Falamaki et al., 2004; Yoshino et al., 2005; Bouzerara et al., 2006; Wang et al., 2006; Nandi et al., 2008; Kim and Van der Bruggen, 2010). In the present research, aimed at monitoring the photocatalytic treatment of olive oil mill wastewater in a batch photoreactor containing a catalytic (TiO_2_) membrane, an investigation was made of the principal parameters affecting the water treatment process, such as the variation in concentration between the beginning and the end of the photocatalytic process of compounds selected as indicators, i.e., polyphenols, or COD variation. In addition, an assessment was made of the amount of catalyst, the presence or absence of oxidizing agent and the varying power of the UV radiation used for photocatalytic irradiation by performing various tests on mill wastewater samples. The catalytic process was monitored by periodically taking samples from the reactor and analyzing them in order to determine both the total polyphenol content using a tyrosinase enzymatic biosensor previously developed by the authors (Campanella et al., 2005, 2006, 2007), and the COD (Chemical Oxygen Demand), measured colorimetrically by the dichromate method (Rand et al., 1979).

## Experimental section

### Materials

Raw materials for ceramic membranes such as clay, kaolin, feldspar, quartz, calcium carbonate and sodium carbonate were provided by Imerys Minerali SPA, Avezzano, Italy. Polysulfone, PVDF (Polyvinylidenefluoride), 1-octanol and N-methyl-pyrrolidone (NMP) was purchased from Sigma Aldrich srl, Milan, Italy. The metallic membrane was supplied by SETEC srl, Civita Castellana, Italy. Titanium oxide (P25) from Evonik Degussa GmbH, Frankfurt am Main, Germany. COD was determined using test kits (LCI 400 CSB/COD/DCO9) supplied by Hach Lange, Dusseldorf, Germany. The tyrosinase enzyme for the biosensor from mushroom (2400 Umg^−1^) (EC 1.14.18.1) and the dialysis membrane (D-9777) were supplied by Sigma, St. Louis, Mo, U.S.A. Potassium chloride, phenol, phosphate for the buffer, potassium dichromate and the other chemical reagents were of analytical reagent grade and supplied by Carlo Erba, Milan, Italy. Lastly, the Kappa-carrageenan was from Fluka AG, Buchs, Switzerland.

### Samples

The OMW samples were provided by a three-phase olive oil mill company located in Lazio, Italy. In Table 1 the principal characteristic of the studied samples are summarized.

**Table 1:** **Properties of OMW samples used in this study**.

COD, (g/L)	47
Total Phenols (TPh), (g/L)	8.1
pH	4.6
Color (absorbance at λ =450 nm)	Dark brown

Properties of OMW prior to dilution.

### Apparatus

**Figure 1:**
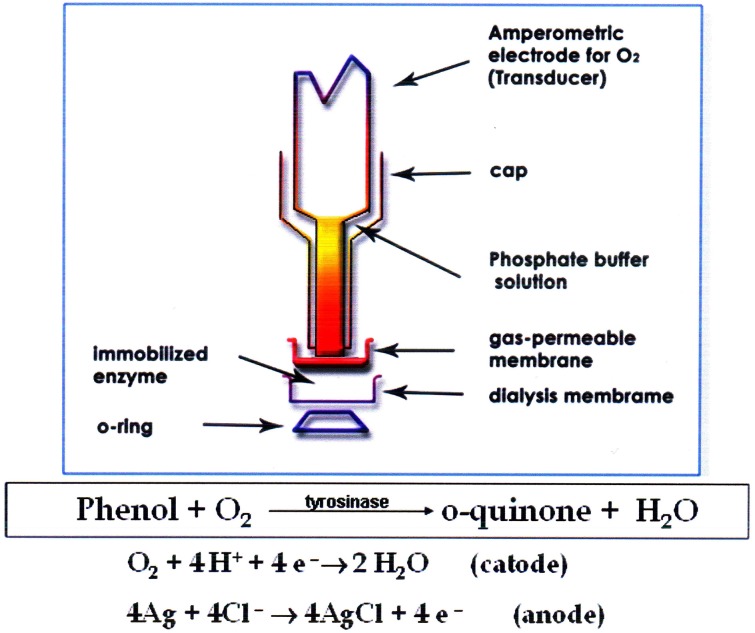
**Tyrosinase enzyme biosensor**.

A tyrosinase biosensor previously developed by the authors (Campanella et al., 2005, 2006, 2007), (see Figure 1) was used for Total Phenols (TPh) determination. The tyrosinase biosensor was assembled using an amperometric oxygen electrode by Universal Sensor Inc., New Orleans (U.S.A.), Mod. 4000-1, connected to a mod. 551 VA-Detector Amel potentiostat coupled to a Mitel MK 5001 Multimeter and to a mod. d5126-2 Omniscribe analog recorder. Total Chemical Oxygen Demand (Rand et al., 1979) was determined using a Hach DR 6000 Spectrophotometer (Hach Lange, Dusseldorf, Germany) and a LT 200 Thermostat for standard and special digestions. A batch type photoreactor designed for laboratory scale analysis was utilized. The bench-scale OMW experimental setup consisted of an aerated photoreactor and a side-stream tubular membrane module. The aerated bioreactor was made of glass and had an operating volume of 2 litres, a diameter of 10 cm and length of 55 cm. Air was introduced into the reactor using filtered in–house compressed air via air diffusers placed at the bottom of the reactor. UV irradiation was provided by a 450 W high pressure Hg lamp, emitting radiation in the 300–400 nm range, or with a 36 W low pressure lamp, emitting radiation in the 250–300 nm range, both produced by Helios Italquartz, Milan, Italy.

### Photocatalysis

Photocatalytic processes make use of a semiconductor metal oxide (TiO_2_) as catalyst and H_2_O_2_ as oxidizing agent. The mechanism of photocatalytic oxidation of organic compounds is well know (Hoffmann et al., 1995; Linsebigler et al., 1995; Herrmann et al., 1999; Gelover et al., 2004; Chatterjee and Dasgupta, 2005; Fostier et al., 2008) and involve multiple processes. Initially, an electron–hole (e^−^/h^+^) pair is generated in the semiconductor particles (Hoffmann et al., 1995), when the surface is irradiated with energy (greater than) or equal to the band gap. Electrons are excited from the valence band (VB) to the conduction band (CB) of the semiconductor, thus creating an electron vacancy at the VB edge. The VB hole is strongly oxidizing, whereas the CB electron is strongly reducing. A hole can migrate to the surface and oxidize an electron donor; in turn, at the surface, the semiconductor can donate electrons to reduce an electron acceptor. Consequently, the semiconductor particle can act either as an electron donor or an electron acceptor for molecules in the surrounding medium, depending on the charge transfer to the adsorbed species (Linsebigler et al., 1995; Chatterjee and Dasgupta, 2005). Overall, the mechanism of photocatalysis can be divided into five steps: (1) transfer of reactants in the fluid phase to the surface; (2) adsorption of the reactants; (3) reaction in the adsorbed phase; (4) desorption of the products; and (5) removal of products from the interface region (Herrmann et al., 1999). A photocatalyst is a substance that, after being irradiated by light, can induce a chemical reaction in such a way that the actual substance of the catalyst will not be consumed (Gelover et al., 2004). Among the various types of photocatalyst, TiO_2_ is the most commonly used semiconductor photocatalyst in the conversion of organic pollutants into harmless substances (Fostier et al., 2008). It is known to be relatively inert, corrosion resistant, and less toxic and cheaper (Madhusudan Reddy et al., 2003) than other photocatalysts such as ZnS, WO_3_, etc. Several catalysts have been tested so far (Do et al., 1994; Sayama and Arakawa, 1997; Valden et al., 1998; In et al., 2007), although TiO_2_ in the anatase form seems to have the most appropriate attributes such as high stability, good performance and low cost (Yin et al., 2001; Andersson et al., 2002).

### Three different catalytic membranes (ceramic, polymeric and metallic), fabrication

Substrates cover a wide range of ceramic, polymeric, glass and metallic materials (particularly stainless steel).

#### Ceramic membrane preparation

For the fabrication of the ceramic membrane, capable of liquid waste treatment, several inorganic raw materials were used: clay (19%W/W), kaolin (33%W/W), feldspar (16%W/W), quartz (20%W/W) calcium carbonate (10%W/W), sodium carbonate (2%W/W). The various raw materials used in the fabrication of inorganic membranes had different functional attributes. Kaolin and clay gave the membrane low plasticity and high refractory properties. Sand contributed to the mechanical and thermal stability of the membrane. The porous texture of the ceramic was regulated by calcium carbonate which, under sintering conditions, dissociates into CaO and releases CO_2_ gas. The path taken by the released gaseous CO_2_ created the porous texture of the inorganic membrane and contributed to membrane porosity during the sintering process. Sodium carbonate acted as a colloidal agent and improved the dispersion properties of the inorganic precursors, thereby enhancing the homogeneity of the membrane structure. Likewise, feldspar acted as binder by creating silicate bonds among the elements to increase the mechanical strength of the ceramic membrane. The particle size distribution analysis of the ceramic body was checked using a Malvern Mastersizer 2000 laser granulometer during the membrane fabrication process. The data indicate that almost 90% of the particles had a diameter of less than 10 μm. The average particle size of kaolin, calcium carbonate and quartz was 2.37, 4.11, and 8.40 μm, respectively.

For fabricating this ceramic membrane these raw materials were mixed and a body slip prepared (Almandoza et al., 2004; Saffaj et al., 2004, 2006; Ersu and Ong, 2008; Palacio et al., 2009; Jeonghwan and Van der Bruggen, 2010). Lastly, the dry ceramic membrane was fired at 950°C. The paste was then cast over a gypsum mold of a circular cylinder (55 cm diameter and 5 cm thickness) (Chin et al., 2006). Subsequently, the ceramic body was de-molded and the circular cylinder dried at room temperature for 24 h. After that it was maintained at 100°C for 12 h in a hot air oven. Then the membrane was sintered for 5 h as follows: 1 h to attain 250°C, 3 h to attain 600°C, 1h to attain 900°C and 1 h at a constant temperature of 900°C. During the transition from 100°C to 250°C, a low heating rate was maintained in order to eliminate the induction of thermal stresses caused by the loss of moisture. Subsequent cooling of the membrane was conducted by an atmospheric cooling procedure adopted by switching off the muffle furnace that had previously been maintained at the established sintering temperature. After sintering, membranes had a hard, rigid and porous texture. Finally, the fabricated membrane was polished with silicon carbide abrasive paper (C-220) to obtain a membrane 53 cm in diameter and 4.5 cm thick. The internal surface of the cylinder was glazed with an enamel containing also 10% (w/w), or 30% (w/w), of TiO_2_, then fired again at 700°C. Physical characterization of the membrane was performed after firing. The open porosity of the membrane was evaluated using the water percentage absorption method. Chemical (i.e., corrosion) resistivity of the membrane was evaluated by subjecting the membrane to concentrated HCl and concentrated NaOH solution. Analysis of the membranes before and after the corrosion test was performed to detect any change in elemental composition. The mechanical strength of the membranes was tested using a three point bending load method (ASTM Standard C1211-02, 2003; JIS R 1601, 2008). All these quantitative tests were performed on the ceramic sample prepared for evaluating general membrane performance and characteristics.

#### Polymeric and metallic membrane preparation

To fabricate the polymeric membrane (Legrini et al., 1993; Molinari et al., 2001; Addamo et al., 2004; Cao et al., 2006; Damodar et al., 2009) a homogeneous polymeric solution was prepared containing a polymer, a solvent and an additive such as commercial polysulfone, 1-octanol and NMP, respectively, in addition a 10 or 30 percent by weight of TiO_2_. In practice the alcohol guaranteed a sufficiently fast phase separation in the water containing the coagulation bath. In order to achieve the desired structure of the membrane, PVDF (Polyvinylidenefluoride) with TiO_2_ was used, as reported in literature (Byrne et al., 1998; Molinari et al., 2002). Casting solutions were prepared by mixing 10 wt.% polyvinylidene fluoride (PVDF), with different amounts of nanosize (20 nm) Degussa P25 TiO_2_ catalyst particles (10 or 30 W/W%) in n-methyl-2-pyrrolidone (NMP) solvent at 60–65°C. The solution containing different percentages of TiO_2_ was sprayed on to a PVC support. After the spraying step, the membrane was immediately exposed for 10 s to a (450 W) high pressure UV lamp to anchor the solution on the PVC support, which was then immersed in a 23–25°C tap water coagulation bath for 1 day. Finally the PVDF/TiO_2_ composite membrane obtained was washed with distilled water.

Lastly, the metallic membrane was made by electro-depositing titanium (Ti) on the surface of a stainless steel foil support (Delplancke and Winand, 1988; Fernandez et al., 1995; Esplugas et al., 2002; Yanga et al., 2004). The stainless steel foil was cleaned by sonication in acetone and placed in a suspension of TiO_2_, Degussa P-25 (mainly anatase) in acetone (1 g in 100 mL). The suspension was first homogenized by sonication and stirred magnetically during the deposition. A counter-electrode of platinum was placed in the suspension just before the stainless steel substrate having the same size and shape. An appropriate potential was applied between the electrodes, the stainless steel foil acting as the cathode. Two samples were prepared by applying 200 V for 4 min. Due to their natural surface charge, the titanium particles moved to the stainless steel foil, thus forming a layer. Finally, to improve adhesion, the sample was heated in a N_2_ flow at 973 K for 4 h to produce the sample TiO_2_/steel. After anodic oxidation, the metallic membrane was rinsed with distilled water and dried in an oven at 40°C (Delplancke and Winand, 1988; Fernandez et al., 1995; Esplugas et al., 2002; Yanga et al., 2004).

### Olive mill wastewater degradation process in a photoreactor

**Figure 2:**
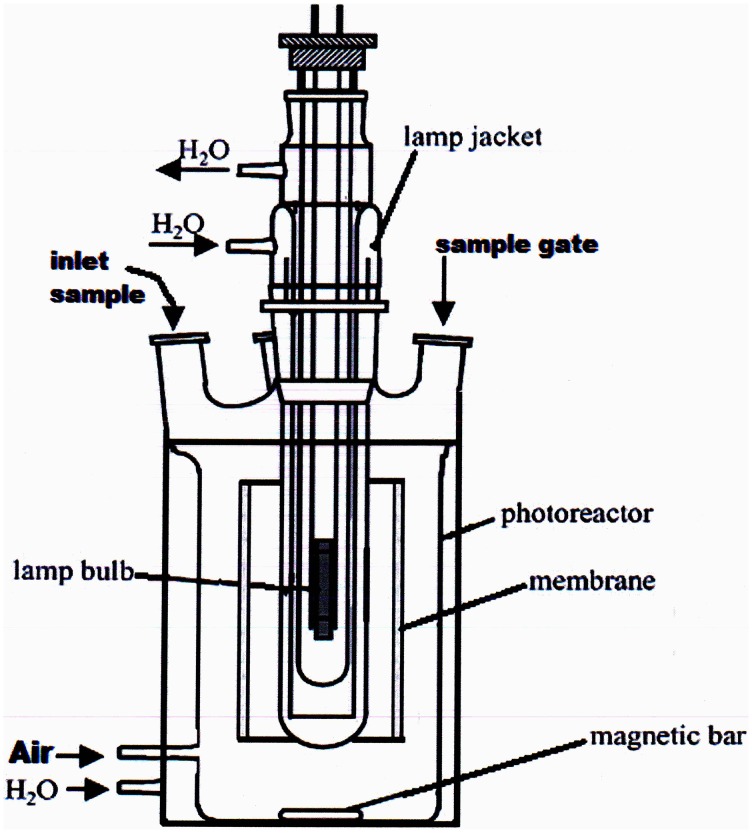
**Scheme of the batch photoreactor (1.5 L) with the membrane placed around the bulb of the lamp**.

Experiments were conducted in a batch type laboratory scale photoreactor, as illustrated in Figure 2. The aerobic olive mill waste water photoreactor (AOP) consisted of a cylindrical vessel with a diameter of 8.0 cm. The working volume was 1.5 L, with provision made for UV irradiation and addition of hydrogen peroxide (the final concentration of H_2_O_2_ in the photoreactor was 5 mM). The catalytic ceramic membrane was mounted as required around the UV lamp, so that the TiO_2_ present in the internal glazed surface was exposed to UV irradiation to achieve the degradation of pollutants. For the sake of comparison, in addition to this new type of ceramic membrane, also two other traditional membranes, either polymeric or metallic, were used alternatively in the same way. In a typical heterogeneous photocatalytic test, 300 mL of OMW were diluted 1:100 V/V with distilled water. Then 1.5 L of the diluted sample were placed in the reactor after adding 4 mL of H_2_O_2_ (0.20 M) in order to obtain a concentration of about 5 mM in the photoreactor and irradiated with a low or high pressure UV lamp. Periodically 5 mL of the sample was taken and their polyphenol content determined using a tyrosinase enzymatic biosensor, fabricated as described in previous papers (Campanella et al., 2005, 2006, 2007), while the COD was determined using the dichromate method (Rand et al., 1979).

## Results and discussion

**Figure 3:**
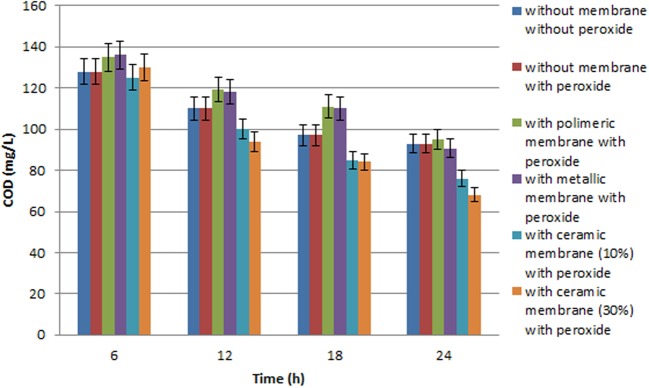
**Chemical oxygen demand variation during batch experiment with the three different membranes used with olive mill wastewater (diluted 1:100 with distilled water), performed with a (450 W) high pressure UV lamp**.

**Figure 4:**
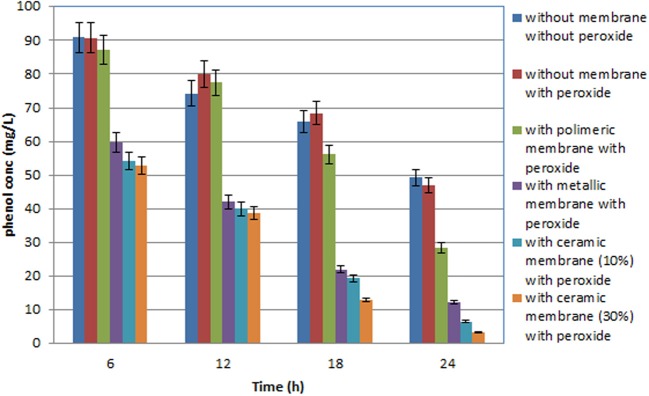
**Total phenolic compound variation in the OMW during batch experiment with the three different membranes used with olive mill wastewater (diluted 1:100 with distilled water), performed with a (450 W) high pressure UV lamp**.

Some papers reported in literature describe the use of microporous membranes for waste water treatment (Di Serio et al., 2008; Cui et al., 2011; Athanasekou et al., 2012) coupled to the catalyst, and the immobilization of TiO_2_ both physically deposited on the membrane surface, or confined in coating. In the present research our aim was to study the use of a ceramic membrane in which 10% W/W, or 30% W/W, TiO_2_ was included in the glaze that cover the internal surface of the tubular membrane. This ceramic membrane had both a filtration and a support function. A new inorganic formulation was also tested for the fabrication of this ceramic membrane, which had an average particle diameter varying from 0.5 to 50 μm. In addition, the present research indicates that the ceramic membrane was fabricated with high contents of inexpensive kaolin (33%) and clay (19%). Thermal characterization suggested that the appropriate sintering temperature for the composition of materials selected is around 900°C. The membranes provided good mechanical strength (3 MPa flexural strength) and chemical stability (only 8% weight loss was found, if immersed in both acid or base media). The results obtained in all the tests performed using the UV lamp at high pressure, expressed in mg L^−1^, are illustrated in the histograms in Figures 3, 4, while all the principal data obtained using UV lamp at low and high pressure, expressed in % removal of COD and phenols, are summarized in Tables 2, 3 respectively.

**Table 2:** **Percentage removal of COD and phenols with a (36 W) low pressure UV lamp after 24 h**.

**Experimental condition**	**% COD removal RSD% ≤ 5.0**	**% phenols removal measured with tyrosinase biosensor RSD% ≤ 5.0**	**pH of the solution after the process RSD% ≤ 5.0**
Without membrane and without H_2_O_2_	28.10	50.2	5.48
Without membrane and with H_2_O_2_	29.05	53.8	5.85
With Metallic membrane and with H_2_O_2_	40.85	82.1	6.95
With Polymeric membrane and with H_2_O_2_	32.52	64.2	7.70
With Ceramic membrane (10 W/W% TiO_2_ on the surface) and with H_2_O_2_	45.95	89.1	7.25
With Ceramic membrane (30 W/W% TiO_2_ on the surface) and with H_2_O_2_	52.42	93.3	7.10

**Table 3:** **Percentage removal of COD and phenols with a (450 W) high pressure UV lamp after 24 h**.

**Experimental condition**	**% COD removal RSD% ≤ 5.0**	**% phenols removal measured with tyrosinase biosensor RSD% ≤ 5.0**	**pH of the solution after the process RSD% ≤ 5.0**
Without membrane and without H_2_O_2_	28.50	50.8	5.50
Without membrane and with H_2_O_2_	29.15	53.8	5.95
With Metallic membrane and with H_2_O_2_	44.85	87.1	6.85
With Polymeric membrane and with H_2_O_2_	38.82	71.4	7.80
With Ceramic membrane (10 W/W% TiO_2_ on the surface) and with H_2_O_2_	50.97	93.2	7.25
With Ceramic membrane (30 W/W% TiO_2_ on the surface) and with H_2_O_2_	56.56	96.8	7.18

Several tests were carried out involving the photodegradation process performed in the photoreactor described and the three different membranes were each used successively. In particular, the process was investigated both in the presence of H_2_O_2,_ but without any catalytic membrane, and with H_2_O_2_ and the catalytic membrane. The results obtained point to a moderate improvement due to the use of H_2_O_2_ in the photodegradation process as the percentage of COD removal increases from 28 to 29%, while the percentage polyphenol abatement varies from about 50 to 53%. Lastly the investigated process was carried out using two different UV lamps, one low and one high pressure. In particular, Tables 2 and 3 show the percentage removal of COD and phenols with an UV lamp at low pressure (36 W) or at high pressure (450 W), respectively, after 24 h, in order to monitor the photocatalytic treatment of OMW in a batch photoreactor using a tyrosinase biosensor and COD test. Comparing the data in Tables 2, 3 the results obtained show that the effect on the process trend does not change substantially whichever type of lamp is used. However, in optimal conditions, the percentage polyphenol abatement varies from about 93 to 97% and that of COD from about 52 to 56% on going from the low pressure lamp to the high pressure one. These results show that on going from the low pressure to the high pressure lamp, at constant treatment time, the pollutant load abatement does not vary by more than 5%, while the cost of the materials (high pressure lamps) and the energy consumption (in kWh) are much higher in the latter treatment. It is therefore more economical to use low pressure lamps in this treatment even though this means lower pollutant load abatement.

It was found that the reaction time have an important effect on COD and phenol removal (see the histograms behavior in Figures 3, 4). After 24 h and in the presence of TiO_2_, using the low pressure UV lamp, almost 46.0 and 89.1% of COD and phenols were, respectively, removed in this process involving the treatment of OMW using a ceramic membrane glazed with an enamel containing 10% W/W TiO_2_. The use of a metallic membrane, on which titanium dioxide, had been deposited using an electrolytic anodizing process, resulted in a percent COD abatement of 40.9% and a percent phenol reduction of 82.1%. Results also show that treatment efficiency increased with increasing TiO_2_ concentration of about 30% in the ceramic membrane, achieving 52.4 and 93.3% in COD and TPh, respectively. This observation indicates that most of the biodegradable compounds initially present in the wastewater were destroyed or/and less biodegradable intermediates were formed. Finally, in the case of the polymeric membrane the final COD removal was only of the order of 32.5% and that of phenols of the order of 64.2%.

The present research was carried out on suitably diluted OMW samples before photocatalytic treatment in the reactor (as described in the previous section). This allowed both the photodegradation process and the analytical methods employed to monitor pollutant load abatement during the process to be tested more accurately. In practice, the aim was above all to give priority to the study of the analytical aspects of the methods used. In future research, the next stage will be to repeat the same tests on increasingly less diluted samples of OMW up to the point of using non diluted “as is” samples, to investigate if and when the increased sample concentration ultimately has an effect on both the efficiency of the photocatalytic process and the monitoring methods used themselves.

## Conclusions

Data reported in the Tables 2, 3 show how the new ceramic membrane containing TiO_2_ seems to provide an excellent combination of thermal, mechanical and chemical stability in addition to good catalytic characteristics. Among the configurations described in this paper, the membrane photoreactor, which combines the advantages of both classical photoreactors and membrane processes, seems very promising. Photocatalytic degradation can be carried out reasonably quickly due to the high available irradiated surface area of the catalytic particles. This study shows that the ceramic membrane is a good candidate for the removal of pollutants in OMW. Indeed, the performance of polymeric, metallic and ceramic membranes was evaluated and compared as far as and the percentage removal of COD and total phenols was concerned. Results show that the ceramic membrane is very effective in removing both compared with the two other membranes. Lastly, it may be concluded that the use of the tyrosinase amperometric biosensor to monitor this process can be considered practical, useful and cheap in monitoring the photocatalytic process of mill wastewater.

### Conflict of interest statement

The authors declare that the research was conducted in the absence of any commercial or financial relationships that could be construed as a potential conflict of interest.
